# Electrocardiographic Progression From Complete Heart Block to Normal Sinus Rhythm in Lyme Carditis Following Antibiotic Therapy: A Case Report

**DOI:** 10.7759/cureus.90460

**Published:** 2025-08-19

**Authors:** Douni O Roger, Majid Yavari, Mahmoud Khairy, Mark Castellani, George Abela

**Affiliations:** 1 Internal Medicine, Sparrow Hospital/Michigan State University, East Lansing, USA; 2 Cardiovascular Disease, Sparrow Hospital/Michigan State University, East Lansing, USA; 3 Electrophysiology, Sparrow Hospital/Michigan State University, East Lansing, USA; 4 Cardiology, Sparrow Hospital/Michigan State University, East Lansing, USA

**Keywords:** antibiotic, borrelia burgdorferi, complete heart block, ecg evolution, electrocardiogram, lyme disease, pacing, second-degree heart block, tick-borne illness

## Abstract

Lyme disease is a leading vector‑borne illness in the United States, and its geographic range has been expanding into the Midwest, including Michigan. Although Lyme carditis is an uncommon complication, it can produce rapidly progressive atrioventricular (AV) conduction disturbances, including complete heart block, that mimic intrinsic cardiac disease and may lead to unnecessary permanent pacemaker implantation if not recognized. We describe a 19‑year‑old woman with a history of postural orthostatic tachycardia syndrome who presented with headache, nausea, vomiting, palpitations, chest discomfort, and bilateral arm paresthesias. She had recently recovered from an upper respiratory infection but denied rash or focal deficits. On presentation, she was bradycardic and borderline hypotensive, and an electrocardiogram showed complete heart block. Laboratory testing revealed elevated inflammatory markers and cardiac biomarkers. She required a temporary transvenous pacemaker and was admitted for management. Lyme serology returned positive, and intravenous ceftriaxone therapy was initiated. Over several days, her AV conduction improved from complete heart block to first‑degree block and ultimately to normal sinus rhythm. The temporary pacemaker was removed, and she completed a course of intravenous antibiotics at home via a peripherally inserted central catheter. This case illustrates the reversible nature of high‑degree AV block caused by Lyme carditis. Early recognition of the condition in young patients with unexplained conduction abnormalities in tick‑endemic areas enables appropriate antimicrobial therapy and avoids unnecessary permanent pacing.

## Introduction

Lyme disease is the most prevalent vector-borne illness in the United States, with over 62,000 confirmed or probable cases reported in 2022; however, broader estimates suggest that up to 476,000 individuals may be diagnosed and treated annually, highlighting potential underreporting [1]. Lyme disease incidence has been rising steadily in the Midwest, particularly in Michigan, where over 500 confirmed or probable cases were reported in 2022. This trend reflects the continued geographic expansion of *Ixodes scapularis *(blacklegged ticks), especially in the Lower Peninsula and regions with dense forestation and recreational activity [2].

Lyme carditis is a relatively rare complication, affecting approximately 1% of patients with Lyme disease in the United States. Surveillance data from 2001 to 2010 found that 1.1% of reported Lyme cases exhibited cardiac conduction abnormalities. Additional registry‑based research estimates the incidence at about 0.056 cases per 100,000 adults per year, with 0.033 per 100,000 adults requiring pacemaker implantation. Despite its rarity, Lyme carditis disproportionately leads to conduction disturbances, and about 59% of affected patients may require permanent pacing [3-5].

Patients with Lyme carditis and high-grade atrioventricular (AV) blocks, such as second- or third-degree (complete) block or a PR interval greater than 300 ms, should be admitted to the hospital, started on intravenous ceftriaxone, and monitored closely [6-8]. Temporary transvenous pacing is frequently necessary, though permanent pacemaker placement is uncommon. Once AV conduction improves (PR < 300 ms), patients can switch to oral antibiotics to complete a 14-21-day treatment course, with most cases resolving within one week [6-8].

We report the case of a 19-year-old female patient with Lyme-induced complete heart block who showed marked clinical and electrocardiographic improvement following treatment with ceftriaxone.

## Case presentation

A 19-year-old female patient with a history of postural orthostatic tachycardia syndrome (POTS) and anxiety presented to a local emergency department in Okemos, Michigan, with a two-day history of headache, nausea, vomiting, and palpitations. She also reported a sudden onset of chest discomfort and bilateral upper extremity paresthesias, which were preceded by headache. She had recently recovered from an upper respiratory infection that resolved approximately one week prior to presentation.

On examination, the patient was alert but appeared mildly uncomfortable. She had no rash, focal neurological deficits, or signs of meningismus, though she continued to endorse subjective paresthesias in both upper extremities. Vital signs were notable for bradycardia with a heart rate of 41 beats per minute and borderline hypotension (BP 105/70 mmHg); respiratory rate was 19 breaths per minute, temperature 36.8°C, and oxygen saturation 99% on room air.

The patient’s initial laboratory evaluation showed elevations in inflammatory and cardiac biomarkers. Key results are summarized in Table 1. Chest X-ray showed no significant abnormality, and the echocardiogram was normal with a left ejection fraction of 60%-65%. An electrocardiogram revealed complete heart block. Cardiology was consulted, and dobutamine therapy was initiated to support cardiac output. The patient was then transferred to the cardiac catheterization laboratory for placement of a temporary transvenous pacemaker and subsequently admitted to the cardiovascular intensive care unit for ongoing monitoring and management.

**Table 1:** Initial laboratory findings *Reference ranges may vary slightly between laboratories.

Test	Patient's value	Typical reference range*	Interpretation
C‑reactive protein (CRP)	6.9 mg/L	<3 mg/L	Elevated
Thyroid‑stimulating hormone (TSH)	4.11 μIU/mL	0.4-4.0 μIU/mL	Slightly high
Free T4	17.29 pmol/L	10-20 pmol/L	Within the normal range
Brain natriuretic peptide (BNP)	279 pg/mL	<100 pg/mL	Elevated
Troponin I high sensitivity	6 ng/L	0-18 ng/L	Within the normal range
Creatinine phosphokinase (CPK)	43 U/L	26-180 U/L	Within the normal range
White blood cells	9,000 cells/μL	4,500-11,000/μL	Within the normal range
Creatinine	0.93 mg/dL	Men: 0.7-1.3 mg/dL. Women: 0.6-1.1 mg/dL	Within the normal range

**Figure 1:**
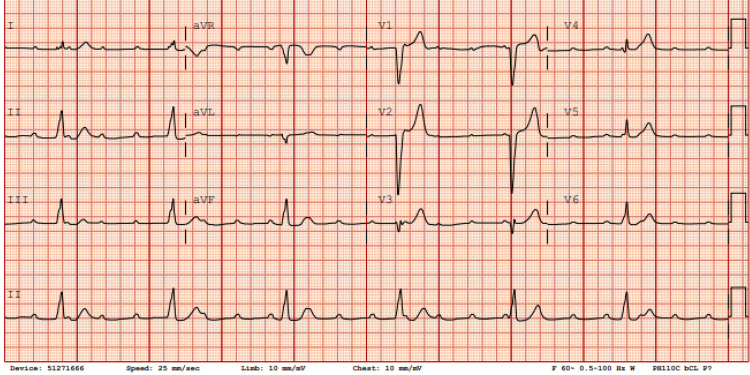
ECG on day 1, prior to antibiotic initiation, showing complete atrioventricular block Lead II shows atrioventricular dissociation; the atrial rate is faster than the ventricular rate.

**Figure 2:**
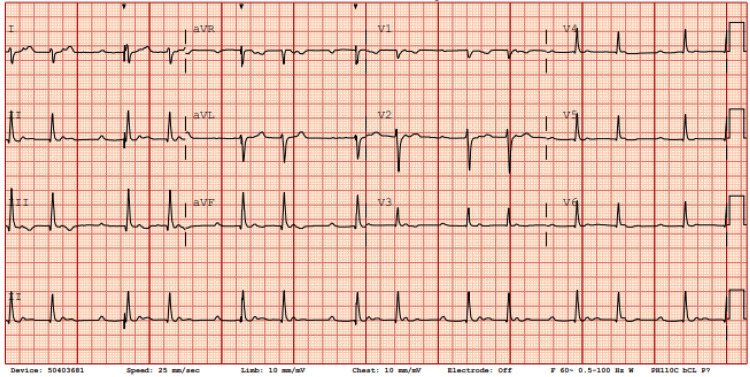
ECG on day 2, one day following ceftriaxone initiation, showing intermittent paced rhythm with a second-degree atrioventricular block, Mobitz I

**Figure 3:**
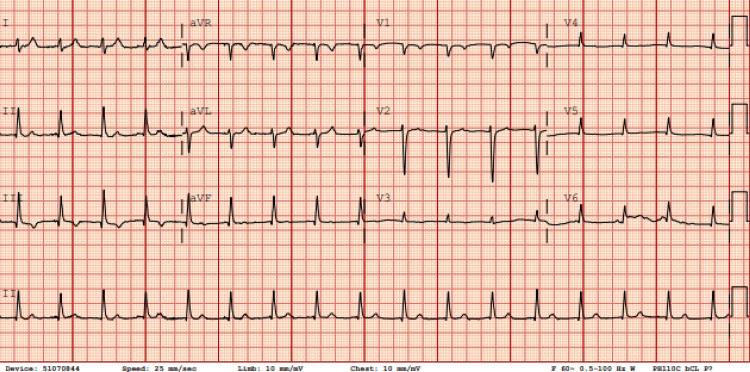
ECG on day 4, three days following ceftriaxone initiation, showing sinus tachycardia with a heart rate of approximately 101 eats per minute Note that every P-wave is followed by QRS.

**Figure 4:**
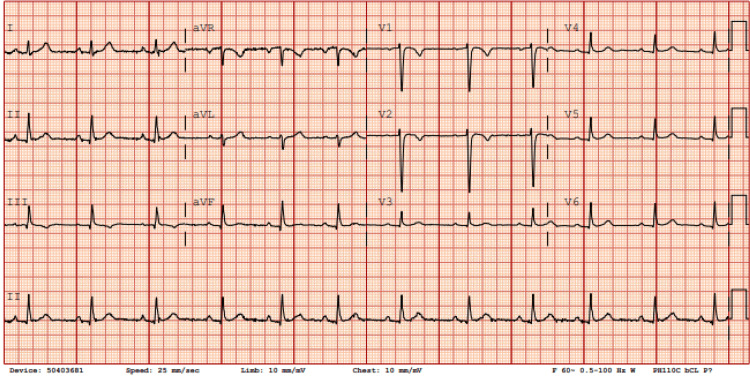
ECG on day 6, five days following ceftriaxone initiation, showing normal sinus rhythm PR interval less than 200 and heart rate of about 70 beats per minute with no evidence of ectopy or conduction block.

Upon admission, the patient was hemodynamically unstable with symptomatic bradycardia; electrocardiography demonstrated complete heart block (Figure 1). A temporary transvenous pacemaker was inserted, and serologic testing confirmed Lyme carditis. A specialist consultant team (infectious disease) recommended a 21‑day course of intravenous ceftriaxone, which was initiated on day 2 of hospitalization. Within 24 hours, her rhythm improved from complete to second‑degree atrioventricular block (Figure 2). By day 4 (approximately 72 hours after starting antibiotics), her rhythm had progressed to sinus tachycardia with marked symptomatic improvement (Figure 3), and by day 6, she was in normal sinus rhythm with complete resolution of symptoms (Figure 4). Electrophysiology supervised the gradual weaning and removal of the temporary pacemaker as her intrinsic conduction recovered. She was discharged home in stable condition with a peripherally inserted central catheter to complete the 21‑day course of ceftriaxone. 

## Discussion

Lyme carditis, though uncommon, is a clinically important manifestation of Lyme disease, often characterized by varying degrees of AV conduction disturbances. This case underscores a classic but often overlooked presentation of high-degree AV block in a young, otherwise healthy individual, a scenario that can easily be misinterpreted as a more serious, intrinsic cardiac conduction disorder. Despite its alarming presentation, Lyme carditis is typically reversible with prompt initiation of appropriate antibiotic therapy. However, the rapid progression and severity of conduction abnormalities may lead clinicians to prematurely implant permanent pacemakers. Our case is especially significant as it documents the complete resolution of complete heart block to normal sinus rhythm within days of starting intravenous ceftriaxone.

Lyme disease follows a well-defined clinical course comprising three stages: early localized, early disseminated, and late disseminated [9]. During the early localized phase, patients commonly present with erythema migrans (EM), a hallmark cutaneous manifestation characterized by a slowly enlarging erythematous lesion, often with central clearing that imparts a classic “bull’s-eye” appearance. This rash is frequently accompanied by nonspecific systemic symptoms, including fever, fatigue, myalgias, and headache [9]. The hallmark of Lyme carditis is rapidly fluctuating AV block, which may progress from mild conduction delays to complete heart block within hours. Patients are often young and otherwise healthy, and symptoms may include lightheadedness, syncope, palpitations, chest discomfort, or dyspnea [10].

Lyme disease is treated based on severity and organ involvement. Early localized infection is managed with oral doxycycline for 10-21 days [1,9]. Lyme carditis with mild conduction delays (PR < 300 ms) can often be treated with oral antibiotics and outpatient monitoring. However, patients with high-degree AV block or PR ≥ 300 ms should be hospitalized, monitored, and treated with intravenous ceftriaxone (2 g daily for 14-21 days) [5]. Temporary pacing may be required for unstable patients, though AV block typically resolves within one week, making permanent pacemakers rarely necessary [5].

The patient initially presented with symptomatic bradycardia and hemodynamic instability due to complete heart block, necessitating placement of a temporary transvenous pacemaker. After serologic testing confirmed Lyme disease, intravenous ceftriaxone therapy was started. Within 24 hours, the conduction improved to a second-degree atrioventricular block, and by 72 hours, the rhythm had progressed to sinus tachycardia with significant symptomatic improvement. By day 6, the rhythm normalized to sinus rhythm and all symptoms had resolved. The patient was discharged with instructions to complete a 21‑day course of intravenous ceftriaxone; overall recovery of the heart block was within six days, consistent with typical timelines reported for Lyme carditis [11].

This case emphasizes the importance of maintaining a high index of suspicion for Lyme carditis in endemic regions and supports a conservative approach to pacing in patients with suspected Lyme-related conduction disease. By capturing the full electrocardiographic evolution and clinical recovery, this report contributes meaningful insight to the literature and reinforces evidence-based strategies to avoid unnecessary long-term device placement.

## Conclusions

This case highlights the reversible nature of high-degree AV block due to Lyme carditis in young individuals, particularly in endemic areas. Early recognition, prompt initiation of intravenous antibiotics, and temporary pacing support were critical to the successful recovery of sinus rhythm. Clinicians should maintain a high index of suspicion for Lyme disease in patients presenting with unexplained conduction abnormalities, especially in the setting of systemic symptoms and recent outdoor exposure during tick season. Timely diagnosis and appropriate antimicrobial therapy can prevent permanent cardiac damage and eliminate the need for long-term pacing.
